# Diagnosis of Subclinical Keratoconus with a Combined Model of Biomechanical and Topographic Parameters

**DOI:** 10.3390/jcm10132746

**Published:** 2021-06-22

**Authors:** Antonio Pérez-Rueda, Diana Jiménez-Rodríguez, Gracia Castro-Luna

**Affiliations:** 1 Department of Ophthalmology, Torrecárdenas University Hospital, 04009 Almería, Spain; a.perezrueda.oft@gmail.com; 2 Department of Nursing, Physiotherapy and Medicine, University of Almería, 04120 Almería, Spain; djr239@ual.es

**Keywords:** subclinical keratoconus, topography, biomechanics, Corvis ST

## Abstract

This study sought to develop a diagnostic model with aberrometry and biomechanical variables for subclinical keratoconus. The design was a cross-sectional study. The topographic data were obtained with a rotating Scheimpflug camera (Pentacam HR), and biomechanical data were obtained with Corvis ST. The study included 81 eyes distributed in 61 healthy corneas and 20 subclinical keratoconus (SCKC), defined as eyes with suspicious topographic findings, normal slit-lamp examination, and a manifestation of keratoconus. Analyses of the topographic and biomechanical data were performed, and a classifying model of SCKC was elaborated. The model for the diagnosis of SCKC includes posterior coma to 90°, Ambrósio’s Relational Thickness in the horizontal profile (ARTh), and velocity when the air pulse is off (A2 velocity). The sensitivity was 89.5%, specificity 96.7%, accuracy 94.9%, and precision 89.5%. The area under the curve (AUC) of the receiver operating characteristic (ROC) curve for the model was 0.951. Diagnosis of subclinical keratoconus depends on the aberrometry variable posterior coma to 90° and the biomechanical variables A2 velocity and ARTh.

## 1. Introduction

Keratoconus (KC) is an idiopathic degenerative eye disease [1] with corneal thinning and a cone-shaped protrusion, which typically occurs in the inferior and temporal zones [2,3,4]. This corneal distortion causes a considerable decrease in the quality of vision because of irregular astigmatism, high myopia and higher-order aberrations [5,6]. It usually appears in youth, progressing into the thirties or forties. Keratoconus incidence and prevalence rates are changing. Recently, Bak-Nielsen et al. [7] reported that the average incidence rate in 2011–2015 was 3.60 per 100,000 person-years. The diagnosed keratoconus prevalence in the Denmark National Patient Register 1977–2015 was 44 per 100,000 persons. These values were higher than those previously reported by Godefrooij et al. [8], who published an annual keratoconus incidence of 13.3 per 100,000 person-years. The estimated keratoconus prevalence was 265 cases per 100,000 persons in the Netherlands. The latest European study conducted in Norway has reported an estimated prevalence of 192.1 per 100,000 and an estimated annual incidence of 19.8 per 100,000 [9].

Although its etiology is unknown, keratoconus has been related to hereditary [10] and environmental factors [11,12]. Biochemical changes in keratoconus corneas, such as increased proteolytic enzymes and decreased inhibitors, have been reported. Even a progressive reduction in collagen produced by keratocytes and a disruption of their organization has been observed to decrease the average diameter of the fibrils and the space between them. A waviness of collagen lamellae have been described. Biomechanical stability depends on the regulation and organization of structural elements of the cornea. Microstructural, biochemical, and cellular alterations negatively affect the structural integrity and develop an abnormal corneal deformation under intraocular pressure measurement [13].

The keratoconus diagnosis is essentially clinical. Subclinical keratoconus (SCKC) is defined as “an eye with suspicious topographic findings (mild asymmetric bow-tie with or without skewed axis and low anterior curvature), normal slit-lamp examination and manifest keratoconus in the fellow eye” [14]. The early diagnosis of keratoconus has been studied using topography, tomography, aberrometry, and biomechanical devices.

The Oculus Pentacam system provides maps with anterior and posterior topography, pachymetry, and aberrometry. The Corvis ST is a non-contact device that provides information about the biomechanical corneal reaction using dynamic Scheimpflug imaging analysis and an air-puff to induce dynamic corneal deformation.

The diagnosis of subclinical keratoconus mainly depends on topographic, aberrometry parameters and biomechanical changes [15]. Therefore, this study aims to derive a classifying model of subclinical keratoconus based on aberrometry and biomechanical variables.

## 2. Materials and Methods

### 2.1. Patient Selection and Study Design

It was a cross-sectional study designed to evaluate the aberrometry and biomechanical variables of SCKC. The data were obtained using a rotating Scheimpflug camera (Pentacam) and a Corvis ST device. Patients with SCKC and healthy corneas were recruited in the Ophthalmology Department at the Torrecárdenas Hospital, Almería, Spain, between January 2020 and January 2021. The data were collected from the Pentacam and Corvis ST clinical databases. The authors declare no conflicts of interest with the manufacturers of Pentacam or Corvis ST. All experiments were performed following the relevant guidelines and regulations. All experimental protocols were approved by the Almeria Research Ethics institutional and licensing Committee (C.E.I./CEIm) located at Torrecárdenas Hospital, with the committee reference number 19/2019. Before the study, participants were informed of the data collected and signed informed consent for their data to be used anonymously. The ethical principles for medical research on human beings from the Declaration of Helsinki were followed.

Eighty-one eyes of 81 patients were distributed as follows:

- Group 1: 61 with healthy corneas. They had the following characteristics: (1) normal topography; (2) negative topographic keratoconus classification; (3) normal biomicroscopy; and (4) no eye disease history. Only one eye per patient was included.
- Group 2: 20 patients with subclinical keratoconus (SCKC). This early stage included patients with (1) minor topographic keratoconus signs and suspicious topographic findings (mild asymmetric bow-tie with or without skewed axis); (2) mean K (mean curvature of keratometry) < 46.5 D; (3) minimum corneal thickness (MCT) > 490 μm; (4) no slit-lamp findings (no central thinning with Fleischer’s ring nor Vogt’s striae); and (5) clinical keratoconus in the fellow eye.

The applied exclusion criteria were a history of any ocular or systemic disease and any ocular surgery, including intracorneal rings segments and corneal collagen cross-linking.

### 2.2. Patient Exam

Patients were examined by the same trained researcher (A.P.R). UCVA and BSCVA were collected with the Snellen chart and logMAR chart. In addition, objective refraction by an autorefractometer (KR8900, Topcon, Japan), biomicroscopy, and eye fundus were examined.

Corneal topography and biomechanics were performed on all patients under the same light conditions and in the central 6.0 mm pupil diameter. Patients with soft contact lenses did not wear them in the examined eye (one for each patient) for 3 weeks, or gas-permeable rigid lenses for at least 5 weeks before the test. The topography was performed using a rotating Scheimpflug camera (Pentacam HR, Oculus Optikgeräte, Wetzlar, Germany) and the biomechanics were assessed with Corvis ST.

The following Pentacam variables were collected: topographic variables of the anterior corneal surface (the flattest curvature of keratometry (K1) and its axis (K1 axis), the steepest curvature of keratometry (K2) and its axis (K2 axis), the mean curvature of keratometry (Km), maximum curvature power on the front of the cornea (KMAX), and the coefficient of asphericity that describes the corneal shape factor (Q)); topographic variables of the posterior surface (the flattest curvature (K1) and its axis (K1 axis), the steepest curvature (K2) and its axis (K2 axis), the mean curvature (Km) and the asphericity (Q)); related pachymetric variables (the central corneal thickness (CCT), the minimum corneal thickness (MCT) with its coordinates (x, y)); related corneal aberrometry variables (the root mean square of total aberrations (total RMS), the root means square of higher-order aberrations (HOARMS) that were calculated up to the third Zernike order for a 6.0 mm pupil diameter, astigmatism to 0° (Z22) and 45° (Z2-2), the anterior horizontal coma to 0°, the posterior horizontal coma to 0°, the total horizontal corneal coma to 0° (Z3¹), the anterior vertical coma to 90°, the posterior vertical coma to 90°, the total vertical corneal coma to 90° (Z3-1), the trefoil to 0° (Z3-3), the trefoil to 30° (Z33), the tetrafoil to 0° (Z44), the tetrafoil to 22.5° (Z4-4), and the spherical aberration (Z40)). In addition, the Belin/Ambrósio Enhanced Ectasia Display (BAD-D) was included.

The Corvis ST variables were as follows: the velocity when the air pulse was on (A1 time, A1 length, and A1 velocity) and off (A2 time, A2 length, and A2 velocity); the highest concavity time (HC time); the maximum deformation amplitude (DA max); the peak distance (PD); and the curvature radius (RHC) at the highest concavity (HC). The Vinciguerra index was also applied, measuring variables such as the maximum deformation amplitude radius at 2 mm and 1 mm, integrated radius, Ambrósio’s Relational Thickness in the horizontal profile (ARTh), and stiffness parameter at first applanation (SP-A1). Central corneal thickness (CCT) was calculated using a horizontal Scheimpflug image at the apex. Intraocular pressure was calculated based on the timing of the first applanation event. It was expressed as the biomechanical corrected intraocular pressure (bIOP). Finally, the Corvis combined biomechanical index (CBI) was measured.

### 2.3. Statistical Analysis

Statistical analysis was performed using the software SPSS Statistics for Windows, version 25.0 (SPSS Inc., Chicago, IL, USA) and R, version 3.5.1. (R core Team, 2018). For each variable, values came from the 3-measurements mean. Therefore, the significance level was *p*-value < 0.05.

Descriptive analyses of demographic characteristics were performed in the 2 groups, with frequencies and percentages for qualitative variables and means and standard deviations for quantitative variables. The Kolmogorov–Smirnov test was performed to check the normality of the variables. Mann–Whitney U tests were performed to compare SCKC patients to the control group because of the non-parametric distribution of the parameters. Finally, correlation between possible confounding factors and topographic and biomechanical variables was calculated with the Spearman test.

According to the forward stepwise entry method (Wald), a binary logistic regression model of SCKC was calculated with tomographic and biomechanical variables. In step 1, the model was built with the variables with statistical differences of the bivariate analysis, and in step 2, the definitive model included the statistically significant variables of the model of step 1. The selection criterion for the proposed model was based on information criteria, specifically, the Akaike information criterion (AIC). The AIC selects the minor complex model (minimum AIC value) with the highest predictability. Furthermore, the variance inflation factor (VIF) of the variables was calculated and indicated the level of collinearity (meaning the correlation or dependence between two or more variables). Then, the Hosmer–Lemeshow test was used as a statistical test for the goodness-of-fit for logistic regression models. All the variables were introduced into the model and were taken out according to their significance. The new model was then contrasted with the previous one; if there were no significant differences, the simplest model with the lowest AIC value, low collinearity (below 2), and best goodness-of-fit was selected.

Finally, the receiver operating characteristic (ROC) curve for the SCKC model was estimated with the area under the curve (AUC). Sensitivity, specificity, accuracy, and precision were calculated with the confusion matrix for model validation. Validation was performed with a cross-validation analysis and bootstrapping technique. Bootstrapping validation is a way to predict the fit of a model to a hypothetical testing set when an explicit testing set is not available. In the cross-validation analysis, the data training instances are partitioned to approximately equal-sized K subsets, each serving as a tuning set and the remaining ones as training.

## 3. Results

This study compared 81 eyes divided into two groups: 61 healthy corneas and 20 SCKC. Demographic and topographic characteristics are shown in Table 1. A comparison between normal and SCKC was calculated. There were statistically significant differences for the variables IOP, anterior coma to 90°, posterior coma to 90°, and BAD-D (*p* = 0.001).

Biomechanical characteristics were measured with Corvis ST and are shown in Table 2. A comparison between normal and SCKC Corvis ST parameters was evaluated. There were statistically significant differences for DA max, A1 time, A1 velocity, A2 velocity, PD, ARTh, SP-A1, and CBI (*p* < 0.05) between the two groups.

The correlations between CCT, IOP, and age and statistically significant topographic (coma posterior to 90°) and biomechanical variables (ARTh and A2 velocity) were analyzed with the Spearman correlation test. There were no statistically significant correlations between age and ARTh (*r* = 0.45, *p* = 0.06), posterior coma 90° (*r* = 0.27, *p* = 0.25), or A2 velocity (*r* = −0.29, *p* = 0.22). There were no statistically significant correlations between CCT and ARTh (*r* = 0.40, *p* = 0.09), posterior coma 90° (*r* = 0.06, *p* = 0.81), or A2 velocity (*r* = −0.15, *p* = 0.53). Moreover, there were no statistically significant correlations between IOP and ARTh (*r* = −0.08, *p* = 0.74), posterior coma 90° (*r* = 0.18, *p* = 0.45), or A2 velocity (*r* = 0.39, *p* = 0.09).

A model to classify patients into SCKC or healthy corneas was determined. A binary logistic regression model was calculated with the R program, version 3.5.1 (R core Team, 2018), including forwarding variables. The dependent variable was the presence of SCKC vs a normal cornea. Every variable with statistical differences in the bivariate analysis (Table 1 and Table 2) was introduced in the first step of the model, except combined index BAD_D and CBI. Variables entered at step 1 were age, IOP ARTh, SP-A1, posterior coma 90°, IOP, anterior coma 90°, DA max, A1 time, A1 velocity, A2 velocity, and peak distance (Table 3).

B is the calculated coefficient for each variable of the model, Exp(B) is the odds ratio for each variable, SE is the standard error, and the Wald hypothesis contrast test is the forward or stepwise selection. The variables entered in step 1 were Ambrósio’s Relational Thickness in the horizontal profile (ARTh), stiffness parameter at first applanation (SP-A1), posterior coma to 90°, intraocular pressure (IOP), anterior coma to 90°, the maximum deformation amplitude (def. amp. max), the time and velocity when the air pulse was on (A1 time, A1 velocity), and off (A2 velocity), and the peak distance (peak dist.). There was statistical significance for ARTh, A2 velocity and posterior coma to 90° (*p* < 0.05) between the two groups.

A hypothesis contrast test comparing both models (Table 4) was calculated, and the result was that there were no significant differences (*p* = 0.1738); therefore, we chose the simplest model with fewer variables.

Table 5 shows the definitive proposed model with the statistically significant variables.

The SCKC model was expressed in the form of an algorithm:

SCKC Index = Logit (SCKC/Normal) = −10.65 − 0.02 (ARTh) + 26.31 (Post Coma 90°) − 56.35 (A2 Velocity)

Logit (SCKC/Normal) is the probability of subclinical keratoconus versus normal; the probability of subclinical keratoconus increases with the posterior coma 90° value and decreases with A2 velocity and ARTh value. A newly diagnosed index named SCKCI (subclinical keratoconus index) was calculated with the model algorithm.

The validation of the model depended on three criteria: the AIC value, which was 40.62 for the proposed model; VIF values under 2 in three variables that indicated their low collinearity; and the Hosmer–Lemeshow goodness-of-fit test. The calculated result was that the regression model was well-calibrated and fitted (*p* = 0.054).

**Figure 1:**
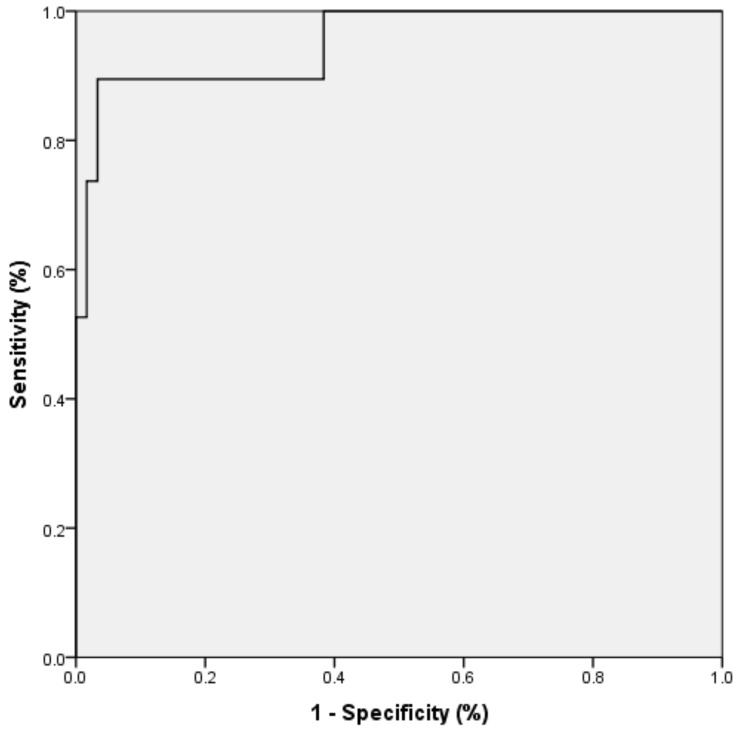
ROC curve for the logistic regression model in SCKC patients.

Table 6 shows the classification table for the proposed model. The sensitivity was 89.5%, specificity was 96.7%, accuracy was 94.9%, and precision was 89.5%. The area under the curve (AUC) of the receiver operating characteristic (ROC) curve (Figure 1) for the binary logistic regression model was 0.951 (CI 95% 0.881–0.998).

**Figure 2:**
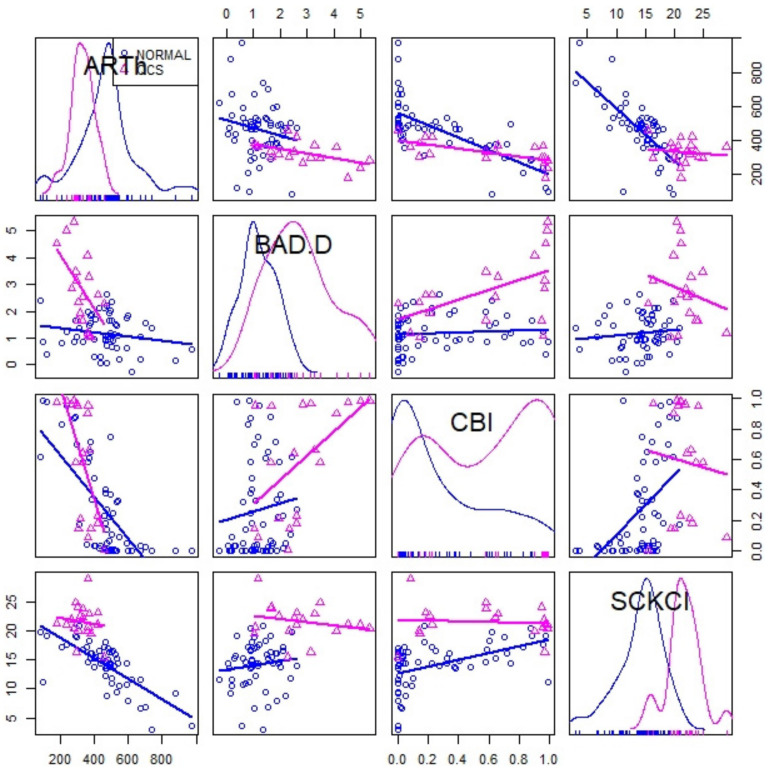
Scattered plot matrix to compare discrimination between normal and SCKC patients.

Figure 2 showed a scatterplot matrix comparing the discriminative capability between normal versus SCKC for standardized indexes as BAD-D, CBI, and ARTh values compared to the proposed SCKCI.

## 4. Discussion

Early diagnosis of keratoconus is necessary to prevent the progression of the disease. Some studies have shown differences between SCKC and normal corneas using topographic, tomographic, and aberrometry parameters, epithelial thickness mapping, and various combinations of indices [16,17,18]. However, others have reported an overlap among the tomographic parameters of these two entities [19,20]. Therefore, biomechanical data are essential to improve the detection of early-stage keratoconus.

There have been several studies on corneal biomechanics using the ORA device and the Corvis ST device [21,22]. However, the Corvis ST is the gold standard in measuring corneal biomechanics. In addition, new Corvis ST parameters have been reported to differentiate SCKC from normal eyes effectively.

Corneal characteristics in SCKC were correlated with decreased viscoelastic structures and corneal stiffness and increased distensibility. Shorter A1 times have been described in SCKC patients than in healthy corneas. In addition, the A1 velocity, the DA_max_, and PD were higher in SCKC patients than in the controls. However, R_HC_ was lower in SCKC than in healthy corneas. Furthermore, A2 time and A2 velocity were higher in SCKC than in healthy corneas, and the A2 length was lower in SCKC [23,24,25,26]. All these variables are consistent with our results shown in Table 3.

Peris-Martínez et al. [27] published a report that A2 velocity was the best parameter to diagnose SCKC patients. According to our results, A2 velocity presented statistically significant differences between SCKC and healthy corneas. Moreover, it was an essential variable in our regression model. In a recent study, Ren et al. [28] showed that ARTh and SP-A1 variables were lower in SCKC eyes than in normal eyes. According to this study, SP-A1 had the highest accuracy in identifying SCKC from control eyes. These results are similar to those obtained in our study. However, the model determined that ARTh for the Vinciguerra index is more significant than SP-A1 and CBI for the diagnosis of SCKC.

The Corvis biomechanical index (CBI) is a parameter provided by the device to differentiate normal patients from established keratoconus. It is calculated by a regression model that includes six biomechanical variables (A1 velocity, ARTh, SP-A1, DA ratio 2 mm, and SD-DA). Vinciguerra et al. [29] calculated a sensitivity of 100%, a specificity of 98.4%, and an area under the curve (AUC) of 0.98 for this parameter to diagnose manifestations of keratoconus. The tomographic and biomechanical index (TBI) differentiates healthy corneas from SCKC. It is obtained by artificial intelligence. According to Ambrósio R. Jr. et al. [30], a TBI of 0.29 provides a sensitivity of 90.4%, a specificity of 96%, and an AUC of 0.822 diagnosing SCKC. Biomechanical coefficients, such as CBI and TBI, have no real physical meaning when used in materials science. Several models for the early diagnosis of keratoconus have been reported in the literature. Castro-Luna et al. [15] presented a model combining minimal corneal thickness, anterior coma to 90°, and posterior coma to 90° with a sensitivity of 75%, a specificity of 96.34%, and an AUC of 0.92 for the diagnosis of SCKC, without including any biomechanical variables. Peña-García et al. [25] reported an SCKC model with three biomechanical parameters (DA max, A1 time, and CCT). Atalay et al. [31] calculated a linear regression model combining corneal hysteresis (CH) and BAD-D with a sensitivity of 87.1%, a specificity of 91.4%, and an AUC of 0.948 for the diagnosis of SCKC. In our study, the SCKCI model included an aberrometry variable (the posterior coma to 90°) and two Corvis ST variables (a speed, the A2 velocity; and a distance, the ARTh). Sensitivity, specificity, and AUC values were higher than those calculated in previous studies. In 2015, Laza et al. [32] established that corneal biomechanical parameters were weakly correlated with topographic parameters in healthy eyes. Our results are consistent with this study.

However, parameters determining the deformation time and recovery are dependent on two variables: IOP and CCT. Hence, IOP, CCT, and mechanical properties simultaneously influence the response of the test. We cannot distinguish the contribution of mechanical properties alone. For example, corneal thinning in KC eyes decreases the biomechanical proprieties, resulting in focal weakening of the cornea. Moreover, Corvis ST tonometry decreases IOP, which lasts for at least 1 h afterwards [33].

Nevertheless, other authors have demonstrated good repeatability of Corvis ST parameters with three (rather than two) standard deviations of the limits of agreement (LOA) [34]. Most of the studies did not take into account the effect of these two variables to perform comparisons. However, Peña-García et al. [25] divided their sample into groups according to differences in IOP and CCT and defined those variables independent of these factors. A2 velocity, DA max, and R_HC_ were parameters that demonstrated the independence of IOP and CCT measurements, and they can be considered robust parameters. Controlling these confounders or choosing robust parameters are necessary for regression analyses. Recent studies have claimed that due to the fast nature of the applied force in the air-puff test, the supposed measured mechanical resistance of the tissue against an air-puff may only come from the inertial effect from the presence of the mass of water [35,36].

There were some limitations to this study. Firstly, the SCKC sample size must be increased in future investigations. However, similar SCKC eyes have been evaluated in previously published studies [25,27,37,38,39] (between 12 and 28 SCKC). The lack of validation in an independent cohort is truly a significant limitation common to all biomedical investigations. We have performed cross-validation (CV) and bootstrapping (BTS) tailored for small sample problems. Both methods have commonly been used to determine such estimates for small-sample classification problems encountered in biomedical applications. Another limitation of the study was the old age of the SCKC sample. Progression at these ages may not be assessable, and biomechanical parameters may be modified. The usefulness at young ages may be questionable. Further extensive and multi-centre studies would be necessary to validate our model in an external population.

## 5. Conclusions

We have proposed a new model for the diagnosis of subclinical keratoconus that depends on an aberrometry variable (the posterior coma to 90°), a biomechanical variable (the A2 velocity), and a biomechanical index (the ARTh).

## Figures and Tables

**Table 1:** Normal vs. subclinical keratoconus. Comparison of demographic and Pentacam variables.

		Mean	Std. Deviation	Std. Error	95% Confidence Interval		Sig.
					Lower Bound	Upper Bound	
Age (years)	Normal	45.85	20.04	2.57	40.72	50.99	0.09
	SCKC	37.2	13.19	2.95	31.03	43.37	
BSCVA (decimal scale)	Normal	0.98	0.05	0.01	0.97	0.99	0.97
	SCKC	0.99	0.07	0.02	0.95	1.02	
Sph Eq *(diopters)	Normal	−1.04	3.16	0.47	−1.98	−0.1	0.52
	SCKC	−1.85	1.6	0.44	−2.81	−0.88	
Q (µm)	Normal	−0.34	0.27	0.04	−0.41	−0.27	0.57
	SCKC	−0.41	0.19	0.04	−0.5	−0.32	
KMAX (diopters)	Normal	45.54	2.07	0.27	45.01	46.07	0.61
	SCKC	46.15	2.12	0.47	45.16	47.14	
CCT (µm)	Normal	529.48	51.08	6.59	516.29	542.68	0.17
	SCKC	511.4	30.04	6.72	497.34	525.46	
IOP (mmHg)	Normal	16.19	3.55	0.45	15.28	17.1	0.00 *
	SCKC	13.6	2.06	0.46	12.64	14.56	
RMS HOA (µm)	Normal	0.51	0.25	0.03	0.45	0.57	0.31
	SCKC	0.63	0.3	0.07	0.49	0.77	
Coma 0° (µm)	Normal	0	0.23	0.03	−0.06	0.06	0.91
	SCKC	−0.05	0.28	0.06	−0.18	0.09	
Anterior Coma 90° (µm)	Normal	0.01	0.21	0.03	−0.04	0.06	0.00 *
	SCKC	−0.5	0.47	0.11	−0.72	−0.28	
Posterior Coma 90° (µm)	Normal	−0.01	0.05	0.01	−0.02	0.01	0.00 *
	SCKC	0.11	0.11	0.02	0.06	0.16	
Sph Ab ** (µm)	Normal	0.19	0.14	0.02	0.15	0.22	0.67
	SCKC	0.15	0.14	0.03	0.08	0.21	
BAD-D	Normal	1.18	0.65	0.08	1.02	1.35	0.00 *
	SCKC	2.64	1.37	0.31	2	3.28	

* Spherical equivalent = spheric refraction + 1/2 cylinder refraction; Sph Ab ** = Spherical aberration; Belin/Ambrósio Enhanced Ectasia Display (BAD-D).

**Table 2:** Comparison of normal vs. subclinical keratoconus Corvis parameters.

		Mean	Std. Deviation	Std. Error	95% Confidence Interval for Mean		*p*-Value
					Lower Bound	Upper Bound	
Def. Amp. Max [mm]	Normal	1.03	0.10	0.01	1.01	1.06	0.01 *
	SCKC	1.13	0.11	0.02	1.08	1.18	
A1 Time [ms]	Normal	7.52	0.39	0.05	7.42	7.62	0.00 *
	SCKC	7.22	0.22	0.05	7.11	7.32	
A1 Deflection Length [mm]	Normal	2.35	0.31	0.04	2.27	2.43	0.24
	SCKC	2.24	0.24	0.05	2.12	2.35	
A1 Velocity [m/s]	Normal	0.14	0.02	0.00	0.14	0.15	0.00 *
	SCKC	0.16	0.02	0.00	0.15	0.17	
A2 Time [ms]	Normal	21.60	1.03	0.13	21.33	21.86	0.08
	SCKC	22.01	0.58	0.13	21.74	22.28	
A2 Deflection Length [mm]	Normal	3.21	0.80	0.11	3.00	3.42	0.68
	SCKC	3.00	0.76	0.17	2.65	3.36	
A2 Velocity [m/s]	Normal	−0.23	0.04	0.01	−0.24	−0.22	0.00 *
	SCKC	−0.27	0.04	0.01	−0.29	−0.25	
HC Time [ms]	Normal	17.03	0.66	0.09	16.86	17.20	0.88
	SCKC	17.12	0.40	0.09	16.93	17.31	
Peak Dist. [mm]	Normal	4.80	0.39	0.05	4.70	4.90	0.00 *
	SCKC	5.10	0.25	0.06	4.98	5.22	
Radius [mm]	Normal	7.11	1.18	0.15	6.80	7.41	0.34
	SCKC	6.75	0.78	0.18	6.38	7.11	
DA Ratio Max (2mm)	Normal	4.61	3.46	0.44	3.72	5.50	1.00
	SCKC	4.60	0.50	0.11	4.36	4.83	
DA Ratio Max (1mm)	Normal	1.68	0.65	0.08	1.51	1.84	0.80
	SCKC	1.61	0.06	0.01	1.58	1.63	
Integrated Radius [mm^−1^]	Normal	8.27	1.38	0.18	7.92	8.62	0.10
	SCKC	9.04	1.34	0.30	8.41	9.67	
ARTh	Normal	481.87	189.43	24.46	432.94	530.81	0.00 *
	SCKC	332.46	67.37	15.46	299.99	364.94	
SP A1	Normal	113.54	18.51	2.39	108.76	118.32	0.00 *
	SCKC	89.61	15.25	3.41	82.47	96.74	
CBI	Normal	0.27	0.32	0.04	0.18	0.35	0.01 *
	SCKC	0.59	0.38	0.09	0.41	0.77	

Ambrósio’s Relational Thickness in the horizontal profile (ARTh); Corvis combined biomechanical index (CBI); stiffness parameter at first applanation (SP-A1). * *p*-value < 0.05.

**Table 3:** Binary logistic regression model. Step 1: variables in the equation.

	B	S.E.	Wald	df	Sig.	Exp (B)
Age (years)	−0.04	0.05	0.96	1.00	0.33	0.96
IOP (mmHg)	1.52	2.68	0.32	1.00	0.57	4.57
Anterior Coma 90 (µm)	0.02	1.93	0.00	1.00	0.99	1.02
Posterior Coma 90° (µm)	40.21	16.52	5.93	1.00	0.01 *	2.899E+17
A1 Velocity [m/s]	−34.52	62.95	0.30	1.00	0.58	0.00
A2 Velocity [m/s]	−98.81	44.43	4.95	1.00	0.03 *	0.00
A1 Time [ms]	−25.29	28.01	0.82	1.00	0.37	0.00
ARTh	−0.02	0.01	4.42	1.00	0.04 *	0.98
Def. Amp. Max [mm]	−27.39	18.15	2.28	1.00	0.13	0.00
Peak Dist. [mm]	−1.32	3.89	0.11	1.00	0.74	0.27
Constant	187.97	181.22	1.08	1.00	0.30	4.293E+81

* *p* < 0.05.

**Table 4:** Hypothesis contrast test comparing models.

	Resid	Dif Resid	Dev	Dif Dev	Pr (>Chi)
Model 1	68	22.35			
Model 2	75	32.62	−7	−10.27	**0.1738** *

* *p* **>** value 0.05. Model 1: SCKC ~ Age+ IOP+ A1.Time..ms. + A1.Velocity..m.s. + A2. Velocity..m.s. + ARTh + PostComa. 90° + Ant. coma.90° + Def.. Amp.. Max..mm. + Peak. Dist...mm. Model 2: SCKC ~ A2. Velocity..m.s. + ARTh + Coma.post. 90°.

**Table 5:** Proposed logistic binary regression model.

	B	SE.	Wald	df	Sig	Exp(B)	95% CI for EXP(B)		
							Lower	Upper	VIF **
ARTh	−0.02	0.01	9.48	1.00	0.00 *	0.99	0.98	1.00	1.33
Posterior Coma 90° [µm]	26.31	8.28	10.10	1.00	0.00 *	2675E+11	24,049.33	2976E+18	1.34
A2 Velocity [m/s]	−56.35	18.11	9.68	1.00	0.00 *	0.00	0.00	0.00	1.38
Constant	−10.65	4.24	6.31	1.00	0.01 *	0.00			

* *p* < 0.05; ** VIF, variance influence factor; B, coefficient; SE, standard error; Wald, forward or stepwise selection (Wald). ARTh, Ambrósio’s Relational Thickness in the horizontal profile; posterior coma 90°, coma posterior to 90°, A2 velocity, velocity when the air pulse is off.

**Table 6:** Logistic binary regression model: classification table.

Observed	Predicted		
	NORMAL	SCKC	Percentage Correct
NORMAL	58	2	96.7
SCKC	2	17	89.5
Overall Percentage			94.9

## Data Availability

The data presented in this study are available on request from the corresponding author.
